# Induction of Systemic Resistance against Sheath Blight in Rice by Different *Pseudomonas* Isolates

**DOI:** 10.3390/life12030349

**Published:** 2022-02-27

**Authors:** Mohsen Mohamed Elsharkawy, Raghda M. Sakran, Abdelmonim Ali Ahmad, Said I. Behiry, Ahmed Abdelkhalek, Mohamed M. Hassan, Amr Ahmed Khedr

**Affiliations:** 1 Agricultural Botany Department, Faculty of Agriculture, Kafrelsheikh University, Kafr El-Sheikh 33516, Egypt; amr3400@yahoo.com; 2 Rice Research Department, Field Crop Research Institute, Agricultural Research Center, Giza 12619, Egypt; raghdasakran@yahoo.co.uk; 3 Department of Plant Pathology, Faculty of Agriculture, Minia University, El-Minia 61519, Egypt; abdelmonim.ali@mu.edu.eg; 4 Agricultural Botany Department, Faculty of Agriculture (Saba Basha), Alexandria University, Alexandria 21531, Egypt; said.behiry@alexu.edu.eg; 5 Plant Protection and Biomolecular Diagnosis Department, ALCRI, City of Scientific Research and Technological Applications, New Borg El Arab City 21934, Egypt; aabdelkhalek@srtacity.sci.eg; 6 Department of Biology, College of Science, Taif University, P.O. Box 11099, Taif 21944, Saudi Arabia; m.khyate@tu.edu.sa

**Keywords:** *Oryza sativa*, *Rhizoctonia solani*, plant-growth-promoting bacteria, defense enzymes, *Pseudomonas* spp.

## Abstract

Sheath blight disease is a fungal pathogen that causes leaf blight in rice plants, resulting in significant yield losses throughout the growing season. *Pseudomonas* spp. have long been used as biocontrol agents for a variety of plant diseases. Four *Pseudomonas* isolates were tested for their ability to promote rice growth and generate systemic resistance to *Rhizoctonia solani*, the causal pathogen of sheath blight disease. In vitro, *Pseudomonas* isolates produced the growth hormone indole acetic acid (0.82–1.82 mg L^−1^). Additionally, seed treatment with *Pseudomonas putida* suspension outperformed *P. brassicacearum*, *P. aeruginosa* and *P. resinovorans* in terms of germination and vigor evaluation. The maximum seed germination of 89% was recorded after seed treatments with a fresh suspension of *P. putida*, followed by 87% germination in *P. aeruginosa* treatment, compared with only 74% germination in the untreated controls. When compared with the infected control plants, all *Pseudomonas* isolates were non-pathogenic to rice and their co-inoculation considerably enhanced plant growth and health by reducing the disease index to 37% and improving plant height (26%), fresh weight (140%) and dry weight (100%). All *Pseudomonas* isolates effectively reduced sheath blight disease incidence, as well as the fungicide carbendazim, which is recommended for field management of *R. solani*. In comparison to untreated control seedlings, treatment with *Pseudomonas* isolates enhanced the production of peroxidase and polyphenol oxidase enzymes and the expression of the *phenylalanine ammonia lyase* (*PAL*) and *NPR1* genes, which could be involved in disease incidence reduction. In conclusion, the use of *Pseudomonas* spp. has been demonstrated to improve rice growth and resistance to *R. solani* while also providing an environmentally acceptable option to the agroecosystems.

## 1. Introduction

Rice is one of the oldest crops, feeding over half of the global population. After wheat, rice is the second major crop in Egypt [1]. To fulfill customer demand, rice production has increased. Rice sheath blight, caused by the fungus *Rhizoctonia solani*, is a serious production restriction in rice-growing regions of the globe. In terms of seasonal production losses of rice, sheath blight disease (SBD) is considered the second most severe disease after blast [2]. As a result of its high epidemic ability, SBD poses a danger to rice production in temperate and tropical rice-growing countries. The pathogen may be found in both soil and water. Furthermore, it releases a phytotoxin that causes most of the disease’s symptoms [3]. The control of SBD has received a lot of attention up till now. There are no commercially feasible disease control techniques available. Chemical control is sometimes extremely costly and research has shown that no resistance to this disease exists in hundreds of germplasms and wild lines evaluated worldwide [4]. Because of the detrimental impacts of the excessive use of agrochemicals, concern for the agricultural environment is unavoidable. Biocontrol is a potential technique to manage phytopathogens, especially with a growing awareness of the need to avoid pesticide use.

Plant-growth-promoting bacteria (PGPBs) are naturally present in the plants’ rhizosphere. Using PGPBs to manage rice diseases has shown to be a successful method over the past two decades [4]. The formation of growth hormones such as indole acetic acid (IAA), atmospheric nitrogen fixation, solubilization of inorganic phosphate, zinc solubilization and ACC deaminase activity are some methods through which PGPBs promote plant development [5,6,7]. They may also help promote plant health and reduce the risk of phytopathogens via various processes, including antagonism, siderophores production, competition and induced systemic resistance (ISR) [8]. Some PGPB strains have also been proven to elicit ISR in plants once applied to seeds or seedlings [9]. A rice endophytic *B. subtilis* suppressed sheath blight disease in gnotobiotic conditions [10]. Plants could establish nonspecific resistance, which is useful against pathogen invasion in addition to basal resistance responses that operate at the site of pathogen infection [11]. By establishing a defensive mechanism known as rhizobacteria-induced systemic resistance, some isolates of non-pathogenic PGPB potentially decrease disease in distal parts of the treated plants [9]. ISR-inducing PGPBs have also been shown to improve the plant’s defense potential by increasing the expression of defense genes [9]. Elevated levels of defense enzymes are recognized to be important in host resistance during ISR. The plant’s defense is activated by PGPB-induced systemic resistance resulting in various defense-related compounds/enzymes in locations far from the pathogen invasion [9].

Peroxidase (class III) is a fast-acting enzyme against different phytopathogens involved in wound healing, lignification, cell-wall elongation and suberification [12,13]. Defense enzymes convert H_2_O_2_ to water and molecular oxygen to protect cells from H_2_O_2_ toxicity during growth [14,15,16]. Polyphenol oxidase (PPO) is critical in the early stages of plant defense when membrane disruption induces the production of phenols such as chlorogenic acid [17]. It reduces free radical formation, which may interact with biological molecules, making the environment unsuitable for pathogen growth. The major enzyme throughout the phenylpropanoid pathway, phenylalanine ammonia-lyase (PAL), is involved in the creation of numerous defense-related secondary chemicals such as phenols and lignin [18,19].

PGPBs have been reported to induce resistance to a variety of plant diseases [20]. Induced resistance in rice, on the other hand, has gained relatively little attention. As a result, the objective of this research study is to determine the efficacy of chosen PGPB *Pseudomonas* spp. for the management of SBD in rice using ISR.

## 2. Materials and Methods

### 2.1. Plants and Pathogen

Rice cv. Sakha 101 was utilized in these experiments. The pathogen *R. solani* was kindly provided by National Research Center, Egypt. Rice plants inoculated with the *R. solni* isolate showed typical sheath blight disease symptoms, such as ovoid or irregular greenish–grey lesions near the waterline or on sheaths and leaf blades [4].

### 2.2. Inoculum Preparation of PGPB Isolates

Four PGPB isolates (*Pseudomonas putida*, *P. brassicacearum*, *P. aeruginosa* and *P. resinovorans*) were obtained from Kafrelsheikh University, Egypt. PGPB were grown on tryptic soy agar at 27 °C for 24 h to ensure purity. The bacterial cells were collected by centrifugation at 10,000× *g* for 4 min. The pellets were resuspended and centrifuged again. After collecting the pellets in sterile distilled water, the concentration was adjusted to 10^8^ cfu mL^−1^ using spectrophotometric measurements (OD _660_).

### 2.3. Characterization of PGPB Isolates

The universal chrome azurol ‘S’ (CAS) test was used to determine the ability of PGPBs to create siderophores [21]. The creation of siderophores was detected by the development of pink color surrounding the bacterial colonies. As a negative control, the non-siderophore-generating bacteria was employed [22]. Bacteria were streaked on LB agar plates with 2% starch, incubated at 32 °C for 72 h and then coated with a layer of Lugol’s solution to detect starch hydrolysis. Starch hydrolysis was shown by the formation of a clear zone surrounding bacterial colonies [23]. IAA generation was identified using a spot test and quantified using HPLC [24,25].

### 2.4. The Impact of PGPBs on Rice Seed Germination and Seedling Vigor

Germination experiments were performed individually for fresh suspensions using the paper-towel technique [26]. Untreated controls and PGPB-treated seeds were sown onto paper towels. One hundred seeds were evenly distributed on germination paper that had been presoaked in distilled water, topped with another presoaked paper towel, then bundled up in polythene wrapping to keep the towels from drying out. The towels were incubated at 24 °C for 14 days. The percentage of germinated seeds was counted. The length of a seedling’s root and shoot were measured to determine vigor [27]. The experiment was performed three times, with 100 seeds for each treatment.

The standard roll-towel technique [26] was used to measure seedling vigor and the vigor index was derived using the formula

Vigor index = % germination × seedling length (shoot length + root length)

### 2.5. The Impact of PGPBs on Rice Growth under Greenhouse Conditions

Rice seeds were surface-sterilized with 2.5% sodium hypochlorite solution and immersed in 5 mL of bacterial suspension (10^8^ CFU/mL) for seed treatment. After 24 h, the bacterial suspension was emptied and the seeds were dried in the shade for 30 min. Seeds treated with PGPB isolates were grown individually in pots packed with sterilized soil, sand and manure (1:1:1 ratio), along with untreated controls. Once a week, each pot received 25 mL of Hoagland’s solution (at 1/3: *v*/*v* strength). Plants grown from carbendazim-treated seeds (4 g kg^−1^ of seeds) were used as a positive control. Seedlings were grown in a greenhouse under controlled conditions, with daily watering and no further fertilizing. Sclerotia of *R. solani* were put in the sheath of the rice plants (two per tiller) 40 days after sowing. The infected area of the plants was covered with absorbent cotton and parafilm was used to secure it. It was then continuously wet with sterile distilled water to keep the humidity high. The severity of the disease was reported and graded from 0 to 5 after seven days [28]. Seedling emergence and growth (plant height, fresh and dry weight) were assessed at 1.5 months after sowing. The experiment was performed three times, with each treatment consisting of 150 seedlings. The disease index was derived using the formula below, which is based on the grades.

Disease index = total grade/no. of sheath observed × 100/maximum grade

### 2.6. Assessment of Defense-Related Enzymes

In this study, the variation in the activity of defense-related enzymes peroxidase (POX; EC 1.11.1.7) and polyphenol oxidase (PPO; EC 1.10.3.1) was determined using enzyme assays at 48 and 72 h post-inoculation (HPI), according to Hammerschmidt et al. [29] and Mayer et al. [30], respectively, and each enzyme test was repeated three times. From rice plants cultivated in the greenhouse (one week after inoculation with the pathogen), 4–5 leaves were chopped into tiny pieces, properly mixed and a 0.1 g sample was processed for an enzyme test right away.

### 2.7. RNA Isolation and qRT-PCR Analysis

After inoculation, the 7th and 8th leaves (from the base to the apex) of the Sakha 101 cultivar were harvested from each replication and treatment at 24 and 72 h (HPI). Leaf samples from non-inoculated plants were also obtained to use as a control. A liquid nitrogen fast freeze was used to freeze the leaves quickly and then the leaves were kept at −80 °C until use. Trizol reagent (Takara, Japan) was used to extract total RNA from frozen samples and DNAse I was used. The total RNA concentration was measured using a NanoDrop 2000 spectrophotometer. The HiScript^®^ 1st Strand cDNA Synthesis Kit (Vazyme, China) was used to synthesize the first strand of complementary DNA (cDNA) for each sample. Analyses were carried out utilizing the 7500 Real-Time PCR System (Applied Biosystem). For the amplification of the two genes mentioned in Table 1, we utilized the primer pairs provided in Table 1. In total, 2 µL of diluted cDNA, 10 µL of Takara’s SYBR Green I Master Mix and 7.2 µL of double-distilled water were used in each reaction. In order to obtain accurate cycle threshold (Ct) values, in total, three biological and three technical duplicates were used for each sample. This experiment relied on a housekeeping gene (*Actin*). Using the comparative ^2−ΔΔ^Ct method, the relative gene expression of the genes in the control sample was determined by setting their expression level to 1 [31].

### 2.8. Field Experiment

The strains were evaluated in the field to see whether they were suitable for large-scale adoption. An endemic area for sheath blight was used to perform two field experiments. The experiment was designed in Randomized Complete Block Design (RCBD) with three replications. For all treatments, a standard plot size of 5 × 5 m^2^ was used. N and P were administered as urea and di-ammonium phosphate (DAP) at 140 and 80 kg ha^−^^1^ (recommended NP), respectively. Positive control was prepared with full/recommended NP with inoculation. Seed treatments were prepared as previously described. The control group consisted of seeds that had just been soaked in distilled water. The treated seeds were planted in a nursery bed after sprouting. Seedlings of 25 days old were manually transplanted in 20 × 20 cm spacing between hills and rows, at the rate of 4 seedlings/hill. The effectiveness of *Pseudomonas* strains was compared to plants developed from a seed treatment with carbendazim (4 g kg^−1^ seeds). The field received the recommended fertilizer dosage. Forty days after planting, the natural occurrence of sheath blight was documented in each plot. The plants were harvested when they reached their full potential. The plants were hand-harvested, sun-dried and weighed. The whole plot’s grain and straw yields were recorded. At the maximal tillering stage, several growth measures, such as plant height and the number of tillers per hill, were recorded. For each treatment, the grain yield was measured at the time of harvest. At the harvest stage, panicles of five random hills from each plot were counted then converted to the number of panicles/m^2^. Ten panicles were randomly collected from each plot to determine the number of filled grains/panicles and 1000 grain weight (g). For grain yield, six inner rows from each plot were harvested, dried, threshed, and the grain and straw were determined. Then, yields/ha were calculated at 14% moisture content.

### 2.9. Statistical Analysis

The analysis of variance (ANOVA) was used to examine the differences among the data collected in the lab, greenhouse and field experiments (XLSTAT Software version 2021.5). The significance at *p ≤* 0.05 was used to assess the importance of PGPB treatment outcomes. Fisher’s LSD differentiated between different types of treatment.

## 3. Results

### 3.1. Characterization of PGPB Isolates

Siderophores were produced by all bacterial strains (Table 2). *Pseudomonas putida*, as well as *P. aeruginosa*, were reported to have the highest production (0.4 mg L^−1^). All isolates were able to hydrolyze starch. *P. resinovorans* showed the lowest ability to hydrolyze starch compared with other isolates. In a spot test, all of the bacteria examined generated IAA, as shown by the pink hue. *P. putida* generated the largest quantity of IAA (1.82 mg L^−1^) followed by *P. aeruginosa* (1.79 mg L^−1^), *P. brassicacearum* (0.89 mg L^−1^) and *P. resinovorans* (0.82 mg L^−1^).

### 3.2. PGPB Effect on Seed Germination and Seedling Vigor

On rice seeds/seedlings, none of the PGPB strains showed any phytotoxic impact. Rice seedlings treated with PGPB strains showed improved growth and resistance against the sheath blight disease. The seed germination percentage of control seedlings was 74%. The germination rate of seedlings treated with PGPB was from 83 to 89%. Seedlings treated with fresh suspensions had a vigor index of 1756–1496, seeds treated with *P. putida* had a vigor index of 1756 and seeds treated with *P. resinovorans* had a vigor index of 1496, compared to 911 in the control group (Table 3). Seeds treated with *P. putida* had the greatest germination rate of 89% and the highest vigor index among the four PGPB strains tested, followed by *P. aeruginosa* with a germination rate of 84% (Table 3).

### 3.3. PGPB Effect on Rice Growth under Greenhouse Conditions

In general, as compared to the untreated controls, all PGPB strains examined exhibited favorable growth responses under greenhouse conditions. When compared to the untreated control, all strains improved seedling height. Seeds treated with *P. putida* and *P. resinovorans* had the highest seedlings recordings of 69.8 and 66.2 cm, respectively, as compared to the untreated control (55.3 cm) (Table 4). Seed treatment of PGPB strains *P. brassicacearum* and *P. resinovorans* also boosted plant height compared to controls. Seed treatment of PGPB strains, especially strains *P. putida* and *P. resinovorans*, boosted fresh weight and dry weight when compared to controls (Table 4).

### 3.4. PGPB Effect on Sheath Blight Infection

PGPB strains provided different levels of protection against sheath blight disease. In terms of disease control, the *P. putida* strain was statistically superior to other *Pesudomonas* isolates but not better than the use of fungicides. Furthermore, compared to untreated plants, *Pseudomonas*-treated plants had much smaller sheath blight lesions in terms of both length and width. When the tested bacteria were administered as seed treatments, the strain *P. putida* had the lowest disease index (37.1), followed by *P. aeruginosa*, *P. brassicacearum* and *P. resinovorans*, which had 39.9, 49.1 and 50.4 of disease index (Table 4).

### 3.5. Defense Enzymes Stimulation by PGPB Isolates in Rice against R. solani

**Figure 1:**
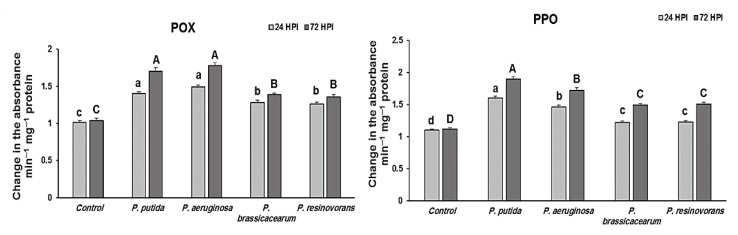
Changes in peroxidase and polyphenol oxidase activities (min^−1^ g^−1^) by different treatments of endophytic *Pseudomonas* isolates in rice plants challenged with *R. solani* under greenhouse conditions. Note: A, B, C, D and a, b, c, d: Different letters represent significant differences.

POX activity began to rise 24 HPI after pathogen inoculation in bacterized rice plants and continued to elevate at 72 HPI in plants treated with *Pseudomonas* isolates. Rice plants treated with *P. aeruginosa* isolates had significantly higher POX than untreated control plants, but the activity was lower than plants treated with *P. putida* (Figure 1). POX activity was likewise up in *P. brassicacearum-* and *P. resinovorans*-treated plants. After challenge inoculation, the maximal activity was seen at 72 HPI in all *Pseudomonas*-treated plants and the activity was sustained at higher levels throughout the experiment. POX activity was lower in plants infected with the pathogen alone (Figure 1). After challenge inoculation, bacterized rice plants challenged with pathogen showed a similar pattern of enhanced polyphenol oxidase (PPO) activity, with activity higher at 72 HPI than at 24 HPI in all *Pseudomonas*-treated plants (Figure 1). In comparison to infected control plants, PPO activity was the highest in the leaves of rice plants inoculated with *P. putida* (Figure 1).

### 3.6. Transcription of NPR1 and PAL Genes in PGPB-Treated Rice Plants

**Figure 2:**
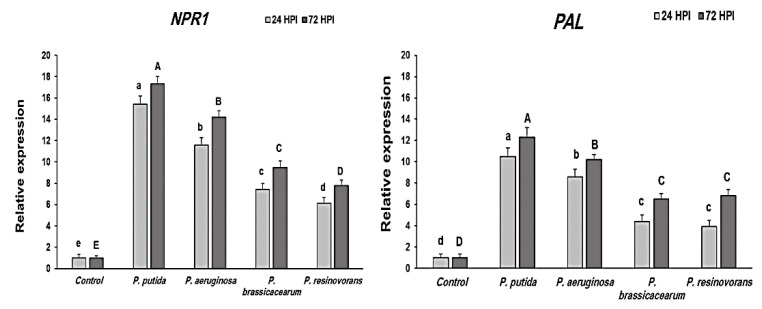
Changes in the relative expression of *NPR1* and *PAL* genes by different treatments of endophytic *Pseudomonas* isolates in rice plants challenged with *R. solani* under greenhouse conditions. Note: A, B, C, D, E and a, b, c, d, e: Different letters represent significant differences.

The results showed that the *PAL* gene, which is involved in JA biosynthesis, is expressed in a constitutive way. However, the relative expression of the gene under appropriate interactions showed considerable variations. *PAL* was highly upregulated as early as 24 HPI (10.5 folds), increasing steadily until 72 HPI (12.3 folds) in the leaves of *P. putida*, which was much greater than *P. aeruginosa*. It was still two times greater than the *P. resinovorans* transcript expression (Figure 2).

Similarly, transcript levels of *NPR1*, a key modulator of salicylic acid, were also considerably elevated in the PGPB-treated plants after 24 and 72 HPI (Figure 2). A considerable increase in the *NPR1* gene was seen in *P. putida*-treated plants during the first 24 HPI (15.4 folds) and continued to increase at 72 HPI (17.3 folds) (Figure 2).

### 3.7. Effect of PGPB Treatments on Plant Growth and Resistance against R. solani under Field Conditions

**Figure 3:**
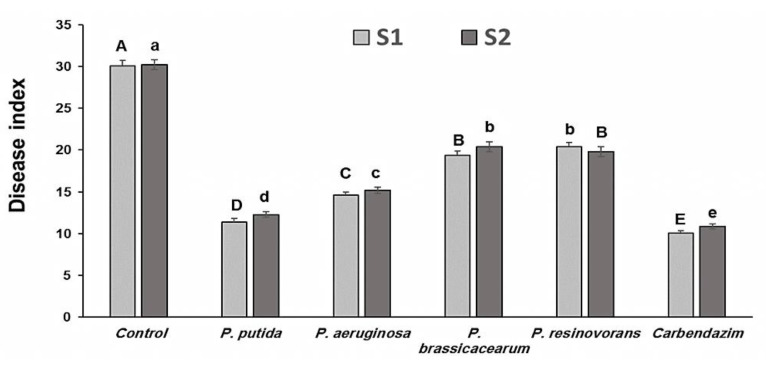
Control of rice sheath blight disease under field conditions by the selected endophytic *Pseudomonas* isolates in comparison to a registered fungicide, carbendazim (4 g kg^−1^ of seeds). S1 and S2 represent the first and second seasons, respectively. Note: A, B, C, D, E and a, b, c, d, e: Different letters represent significant differences.

**Figure 4:**
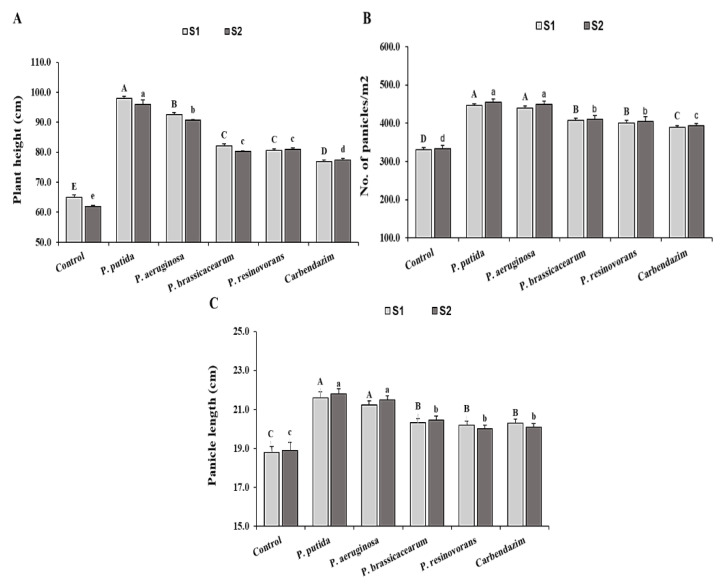
Effect of application of *Pseudomonas* isolates on growth parameters of rice seedlings compared to the controls, under field conditions. Note: A, B, C, D, E and a, b, c, d, e: Different letters represent significant differences. (**A**) Plant height (cm); (**B**) No. of panicles/m^2^; (**C**) Panicle length (cm).

The results from both field experiments revealed that the four *Pseudomonas* isolates performed similarly in two seasons (2018/19 and 2019/20). *P. putida*-applied plots had the lowest disease incidence among PGPB isolates (Figure 3). The regular fungicide treatment had the same effect on disease severity reduction as *Pseudomonas* in both field trials. In the field, improved plant growth was also stimulated (Figure 4). Plant height and number of panicles differed significantly across treatments. In the field experiment, averages of 52 and 32% enhanced plant height were obtained in *P. putida-* and *P. aeruginosa*-treated plots, respectively.

**Figure 5:**
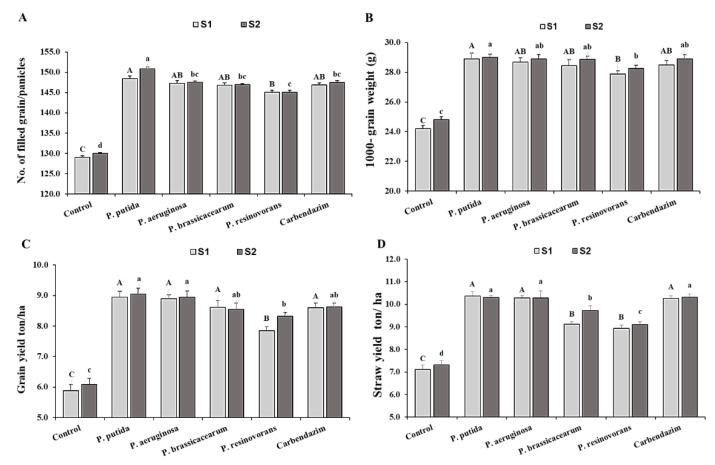
Effect of application of *Pseudomonas* isolates on yield parameters of rice seedlings compared to the controls, under field conditions. Note: A, B, C and a, b, c, d: Different letters represent significant differences. (**A**) No. of filled grain/panicles; (**B**) 100-grain weight (g); (**C**) Grain yield ton/ha; (**D**) Straw yield ton/ha.

In the field trials, rice plants treated with *Pseudomonas* isolates had a significant increase in all growth and yield traits when compared to an uninoculated control (Figure 4 and Figure 5). Seed bio-priming promoted plant growth traits, such as plant height, no. of panicles and panicle length, and yield traits, such as filled grains 1000-grain weight, grain yield and straw, compared with the control in the field experiments (Figure 4 and Figure 5). The results revealed that maximum growth and yield parameters were recorded in the plants bio-primed with *P. putida* and followed by the plants bio-primed with *P. aeruginosa*, fungicide, *P. brassicacearum* and *P. resinovorans.* In *Pseudomonas*-treated plants, the highest grain yield (9 ton ha^−1^) was achieved using *P. putida*, followed by *P. aeruginosa* (8.9 ton ha^−1^) and *P. brassicacearum* and *P. resinovorans*. Although the grain and straw yields of rice plants treated with *Pseudomonas* isolates were not statistically significant when compared to fungicide plots, plant height was significantly higher in *Pseudomonas*-treated plants.

## 4. Discussion

A huge challenge to sustainable rice production in Africa and Asia is fungal diseases. Millions of rice fields are regularly infected in serious epidemics that reduce rice production by more than 60% [32]. PGPB-based biocontrol approaches, which include the treatment of disease-suppressive bacteria to reduce infections and promote plant growth, might be a viable option for managing rice infections. Under greenhouse and field conditions, our findings show that PGPBs were effective in promoting rice growth and control of sheath blight. Plant growth regulators such as cytokinins, gibberellins and indole acetic acid, which may either directly or indirectly control plant growth and development, are produced by *Pseudomonads* isolates [33]. The current study revealed that seed treatment with PGPB isolates increased seed germination and seedling vigor stand in vitro compared to the control. Seeds treated with *P. putida* had the best germination and vigor indexes. In cereals, rhizobacteria have been shown to increase seed germination characteristics in a similar manner [4]. Plant growth may be enhanced by PGPBs either directly or indirectly. Indirect effects are attributed to the synthesis of metabolites such as antibiotics that inhibit the development of phytopathogens and other harmful bacteria. The synthesis of growth regulators is required for the direct effects of PGPBs on plant growth. The PGPBs used in this study were also able to create phytohormone (Indole acetic acid; IAA), indicating that they may be employed to promote plant growth [34]. It is possible that IAA is responsible for growth promotion by PGPBs. IAA helps plants to obtain more water and nutrients from the soil by promoting root development and elongation [35]. IAA has been found to promote plant growth and rhizospheric competence.

Biocontrol is a unique and complex term that includes multiple disease-suppression mechanisms. PGPBs are excellent inducers of resistance against phytopathogens, with certain isolates being effective against a wide range of plant diseases in a variety of crop species. All *Pseudomonas* isolates were able to decrease the incidence of SBD. Among the four isolates of *Pseudomonas* spp., one strain of *P. putida* showed higher levels of disease suppression. The decrease in disease severity resulted in increased plant growth. Plant height and fresh and dry weight were higher in treated plants than in non-treated plants. *Pseudomonas* sp.-induced ISR has been shown to provide rice plants with systemic protection against several diseases [32]. Recognizing these mechanisms would help to ensure that biocontrol agents are used effectively in the field. All of the bacteria tested in this research stud were able to create siderophores. It is well known that siderophores are one of the key processes for the suppression of fungal diseases. These bacteria were also shown to be able to hydrolyze starch. Microorganisms with the capacity to create siderophores have previously been shown to be effective biocontrol agents against plant disease [36]. The host plant was unaffected by any of the PGPB isolates. *Pseudomonas* species are recognized for their ability to combat a wide range of plant pathogens [37]. We noted that the majority of PGPB isolates reduced the severity of SBD. *Pseudomonas* treatments had a dual effect on disease severity and plant development, resulting in higher biomass growth and yield, while, at the same time, carbendazim treatment lowered the disease incidence.

Rice plants infected with the pathogen had significantly higher levels of defense enzymes. POX and PPO activities in plants treated with PGPBs were significantly elevated. Plants treated with *P. putida* had the most elevated PPO activity, whereas plants inoculated with *P. aeruginosa* had the most POX activity. Induced resistance by PGPBs is associated with the increase in defense enzymes [38]. The formation of phenolic compounds in plants in response to infection has long been recognized. POX has a critical part in the production of phenolics, phytoalexins and lignin, the three essential components that contribute to disease resistance [39]. Treatment with *P. fluorescens* increased the POX activity in *Fusarium oxysporum-* and *Pythium*-infected tomatoes, as reported by Ramamoorthy et al. [40]. In this research study, rice seedlings produced from *Pseudomonas* species-treated seeds showed enhanced POX activity after challenge inoculation with the pathogen. Infected plant tissue is oxidized to very toxic quinines by the copper-containing enzyme PPO and this is considered to play a part in the plant’s resistance to disease [41]. It has been shown that cucumber’s PGPB-mediated ISR depends on PPO [42]. In the same manner, as other enzymes, two strains of *Bacillus* exhibited PPO activity against the pathogen. *P. fluorescens* Pf1 increased PPO activity against *R. solani* [43].

*NPR1* and *PAL*, two pathogenesis-related genes, were shown to be expressed at different times in the shoots of rice plants bio-primed with *Pseudomonas* endophytes. Rice plants’ gene expression profiles (also known as mRNA transcripts) were examined in the current research study to see how they changed over time after infection. At 24 and 72 HPI, the relative fold change in the shoots of the treated and control plants was compared to determine the quantitative expression of *NPR1* and *PAL*. *NPR1* and *PAL* were upregulated in the shoot tissues by qRT-PCR. Bio-primed plants with *P. putida* had the greatest *NPR1* expression in the shoot, followed by plants with *P. aeruginosa*. The application of these rhizobacteria as seed treatments could prove to be a beneficial component of integrated pest management. A significant increase in defense gene expressions in treated rice plants under pathogen load was also seen after treatment with PGPB isolates [44]. Phenolic synthesis has been linked to an increase in PAL activity, which protects against diseases. These findings are in line with previous research showing that PAL is a critical enzyme in plants’ induced systemic resistance through the phenylpropanoid pathway. Lignin production was triggered by an increase in PAL, which activated peroxidase [45]. In addition, PAL triggered the production of SA/MeSA and SA-dependent pathways. NPR1-dependent and NPR1-independent pathways are part of the SA-dependent disease resistance mechanism [46]. PR gene expression is involved in the NPR1-dependent pathway, whereas WRKY transcription factors are involved in the NPR1-independent pathway. In plants, these elicitors activate local and systemic resistance (SAR) against numerous biotic stressors via various mechanisms [47]. Plant immune responses are influenced by pathogen species, infection severity and host species. Effector-triggered immunity (ETI) is critical in the biotrophic relationship because it activates the host’s resistance proteins (R-proteins) [48]. Upon detecting pathogen effectors, the R-protein triggers the hypersensitive response (HR). As a result of this immunological response, disease development is highly restricted. These elicitors use different routes to activate local and systemic resistance. The well-known truth is that the proper stimuli or signals are required to activate the defense genes [49].

Application of *Pseudomonas* species to rice seedlings improved the growth and control of SBD by decreasing the severity and increasing the transcription levels of defense genes and enzyme activities. However, the promising biocontrol agents *P. putida* and *P. aeruginosa* need extensive biosafety investigations before they can be released into the environment and used in commercial applications.

## Figures and Tables

**Table 1:** Primers used for gene expression experiment.

Gene Name	Forward Primer Sequence (5′→3′)	Reverse Primer Sequence (5′→3′)	Gene Bank ID
*Actin*	CAGCCACACTGTCCCCATCTA	AGCAAGGTCGAGACGAAGGA	AK058421
*NPR1*	AGAAGTCATTGCCTCCAG	ACATCGTCAGAGTCAAGG	Os01t0194300
*PAL*	GGTGTTCTGCGAGGTGATGA	AGGGTGGTGCTTCAGCTTGT	AK068993

**Table 2:** Growth-promoting determinants and biocontrol of PGPB isolates.

Plant-Growth-Promoting Bacteria (PGPBs)	Siderophores Produced (mg L^−1^)	Starch Hydrolysis	IAA Production (mg L^−1^)
*P. putida*	0.4	+++	1.82
*P. aeruginosa*	0.4	+++	1.79
*P. brassicacearum*	0.3	+++	0.89
*P. resinovorans*	0.3	++	0.82
*Bacillus* sp.	0.1	++	-

*Note:* ++ indicate starch hydrolysis ability of more than half LB agar plates. +++ indicate starch hydrolysis ability of complete LB agar plates.

**Table 3:** Screening of PGPB strains based on vigor index of rice seedlings.

Plant-Growth-Promoting Bacteria (PGPBs)	Germination (%)	Vigor Index
*P. putida*	89 ^a^	1756.4 ^a^
*P. aeruginosa*	87 ^b^	1759.9 ^a^
*P. brassicacearum*	84 ^c^	1544.8 ^b^
*P. resinovorans*	83 ^c^	1496.1 ^c^
Control	74 ^d^	911.6 ^d^

Note: a, b, c, d: Different letters represent significant differences.

**Table 4:** Effect of PGPB strains on sheath blight incidence and plant height under greenhouse conditions.

Plant-Growth-Promoting Bacteria (PGPBs)	Plant Height (cm)	Fresh Weight (g/Seedling)	Dry Weight (g/Seedling)	Disease Index
*P. putida*	69.8 ^a^	1.13 ^a^	0.12 ^a^	37.1 ^d^
*P. aeruginosa*	66.2 ^b^	0.92 ^b^	0.10 ^b^	39.9 ^c^
*P. brassicacearum*	61.6 ^c^	0.73 ^c^	0.08 ^c^	49.1 ^b^
*P. resinovorans*	62.2 ^c^	0.71 ^c^	0.08 ^c^	50.4 ^b^
Carbendazim	54.8 ^d^	0.50 ^d^	0.06 ^d^	31.8 ^e^
Control	55.3 ^d^	0.47 ^d^	0.06 ^d^	68.3 ^a^

Note: a, b, c, d, e: Different letters represent significant differences.

## Data Availability

Not applicable.
